# A comprehensive review of oral glucosamine use and effects on glucose metabolism in normal and diabetic individuals

**DOI:** 10.1002/dmrr.1150

**Published:** 2010-12-07

**Authors:** R R Simon, V Marks, A R Leeds, J W Anderson

**Affiliations:** Cantox Health Sciences InternationalMississauga, ON, Canada; Department of Clinical Biochemistry, Postgraduate Medical School, University of SurreyGuildford, Surrey, UK; School of Biomedical and Molecular Sciences, University of SurreyGuildford, Surrey, UK; Department of Human Nutrition, University of CopenhagenZealand, Frederiksberg, Denmark; Departments of Internal Medicine and Clinical Nutrition, University of KentuckyLexington, KY, USA

**Keywords:** glucosamine, diabetes, glucose, insulin, hyperglycaemia, safety

## Abstract

Glucosamine (GlcN) is a widely utilized dietary supplement that is used to promote joint health. Reports that oral GlcN supplementation at usual doses adversely affects glucose metabolism in subjects with impaired glucose tolerance have raised concerns that GlcN should be contraindicated in individuals with diabetes and those at risk for developing it. This review addresses its potential, when used at typical doses, to affect glucose metabolism and insulin sensitivity in healthy individuals and those with diabetes or ‘pre-diabetes’. Publicly available scientific information and data on GlcN were systematically compiled using the electronic search tool, Dialog®, and reviewed with special emphasis on human studies. In long-term clinical trials, including those containing subjects with type 2 diabetes or ‘pre-diabetes’, GlcN produced a non-significant lowering of fasting blood glucose concentrations in all groups of subjects treated for periods of up to 3 years. Owing to limitations in study design, conclusions based on studies that report adverse affects of GlcN on insulin sensitivity and glucose tolerance in pre-diabetic subjects are suspect. However, no definitive long-term studies of GlcN use for individuals with pre-diabetes are available. Nevertheless, based on available evidence, we conclude that GlcN has no effect on fasting blood glucose levels, glucose metabolism, or insulin sensitivity at any oral dose level in healthy subjects, individuals with diabetes, or those with impaired glucose tolerance.

Copyright © 2010 John Wiley & Sons, Ltd.

## Introduction

Glucosamine (GlcN) is one of the most widely used over-the-counter dietary supplement products for the management of osteoarthritis 1. In a recent US survey, 5% of the general population in the United States reported using GlcN in the previous week, and up to 9% of elderly men and 7% of elderly women were identified as GlcN users 2. Given GlcN's widespread use and popularity, particularly in the elderly, it is expected that a significant percentage of users would have diabetes or pre-diabetes. On the basis of this incidence of GlcN use, and the current prevalence of diabetes in the US population 3, it can be estimated that GlcN is used by almost 400 000 elderly diabetic subjects, and in as many as 2.7 million pre-diabetic individuals. With the ageing population, increasing incidences of diabetes, and increased popularity of dietary supplement use in this population group, the safety of GlcN supplementation in patients with diabetes and those with undiagnosed diabetes warrants consideration as infusion of GlcN in animal models or incubation of GlcN with tissues induces insulin resistance and glucose intolerance.

Mechanistically, it has been hypothesized that the diabetogenic effect of GlcN observed in animal infusion studies is mediated by GlcN augmenting hexosamine biosynthesis in insulin-sensitive tissues, a metabolic pathway that has been implicated in the development of type 2 diabetes 4,5. Recent investigations have reported that oral GlcN consumption in humans worsens insulin resistance in subjects at risk of developing type 2 diabetes 6,7. To date, these observations have not been critically appraised and, as they have raised concerns among clinicians and the general public that GlcN is contraindicated in people with diabetes and those at risk of developing it 8, we have herein critically and comprehensively reviewed the published literature.

## Methods—literature review

A comprehensive package of publicly available scientific data on GlcN in relation to its effect on glucose metabolism was compiled from the literature and other published sources through December 2009. The data were collected using the electronic search tool, Dialog®, consisting of several databases, including MEDLINE®, TOXFILE, AGRICOLA, JICST-Eplus, BIOSIS Previews®, and EMBASE®. To identify all available literature relevant to the safety assessment of GlcN in normal, pre-diabetic, and diabetic individuals, GlcN linked with the following terms were used in the search criteria: diabetes, glucose metabolism, glucose tolerance, glucose intolerance, impaired glucose tolerance, insulin resistance, insulin sensitivity, oral glucose tolerance test, hyperglycaemia, and high blood sugar. All potentially relevant clinical trials, whether controlled or uncontrolled, without regard to route of administrations or duration of exposure, were included in the review.

### Data abstraction

Amongst clinical studies, research papers in which the route of administration was oral were reviewed separately from research papers in which the route of administration was not oral. Moreover, results in diabetic and pre-diabetic subjects were reviewed separately from results in normoglycaemic subjects. Clinical studies in which GlcN was administered orally were summarized in tabular format. The following information was collected for each clinical study: dose, study design, duration, net effects on fasting glucose, and other results relevant to glucose metabolism. The net effect on fasting glucose was calculated by subtracting the change from baseline in fasting glucose in the control group from that in the GlcN group; to permit comparisons between studies, net effects on fasting glucose were expressed as percents.

## Results

### Typical use of GlcN and oral bioavailability

GlcN is mainly sold in one of two commercial forms as a salt of GlcN hydrochloride (GlcN·HCl) or GlcN sulfate (GlcN·SO_4_). When ingested, both salts dissociate fully yielding free GlcN, and the bioavailability of GlcN derived from either source is expected to be the same. GlcN is typically used at a dose of 1500 mg/day (21 mg/kg body weight), but higher doses of 3200 mg/day (45 mg/kg body weight) have been used in some clinical trials 9. Although the limited absorption of orally administered GlcN is not widely acknowledged, it has long been known that the active transport of GlcN in the small intestine does not occur 10. Numerous early studies using *ex vivo* everted sac models and Tris-disrupted brush border preparations (hamster jejunums) have shown that high concentrations of d-GlcN competitively inhibit glucose absorption, and that transport of GlcN is highly inefficient, effects that have been attributed to differences in the equatorial position of the second carbon atom of these sugars 11–13. Recent experiments using Xenopus oocytes expressing various mutant forms of the human sodium-dependent glucose transporter-1, have further confirmed these early observations as the authors show that GlcN is a poor substrate for the transporter, with a *K*_0.5_ value ≫ 100 mM 14.

As the absorption of orally administered GlcN is limited, usual doses result in plasma concentrations in the range of 3–8 µmol/L 7,15–18. The limited bioavailability of GlcN is highlighted by two studies where large oral doses of GlcN were administered: Persiani *et al.* 19 showed that when an oral dose of GlcN·SO_4_ was doubled from 1500 to 3000 mg, there was no significant increase in plasma concentrations of GlcN; and in six healthy volunteers who consumed in excess of five times (7540 mg) typical supplemental doses, plasma GlcN levels measured over a 180-min period did not increase above the detection limit of the assay (15 µmol/L) 20.

Direct measurements of the oral bioavailability of GlcN have been reported for a number of species. The bioavailability of GlcN has been reported to be 10% in dogs 21, in the range of 2.5–6% in horses 22,23, and as high as 20% in rats 24. Furthermore, as reported by Aghazadeh-Habashi *et al.* 24, no differences in the intravenous *versus* intraperitoneal 8-h GlcN plasma concentration–time profiles was observed in male Sprague-Dawley rats administered GlcN at a dose of 350 mg/kg body weight, which indicates that the low bioavailability of GlcN in animals is a function of the limited absorption of intact GlcN in the gastrointestinal tract. The true bioavailability of oral GlcN in humans is unclear although numerous articles cite GlcN bioavailability as 26%. This value was incorrectly derived from the study by Setnikar *et al.* 25, in which the ^14^C label rather than the intact ^14^C GlcN-labelled molecule was measured. No qualitative measurements of intact GlcN were made during this study, and, as shown by Aghazadeh-Habashi *et al.* 24,26, the apparently almost complete absorption of GlcN over the 120-h period reported by Setnikar *et al.* 25 could be attributed to the absorption of bacterial metabolites of GlcN from the large intestine. For example, GlcN is one of the most commonly fermented substrates among human and animal *Bifidobacteria* 27, and numerous *Bacteroides* spp. found in the colon are known to ferment GlcN 28. The limited oral bioavailability of GlcN over a large dose range (1000–7540 mg) is due to its exclusion by the gut and indicates that the liver is not exposed to high concentrations of GlcN in portal venous blood even when consumed at several times the typical amount. Thus, the many observations obtained from animals infused with GlcN, and following the exposure of cells to high concentrations of GlcN in culture in which concentrations of GlcN in the millimolar rather than in the micromolar range are used, must be interpreted with caution.

### GlcN infusion

High plasma GlcN concentrations, which can only be achieved using intravenous infusions, produce insulin resistance and impair glucose tolerance in a number of species, including rats, dogs, and sheep 29–39. During the comprehensive literature search, two studies reporting the effects of GlcN infusion on glucose tolerance and insulin sensitivity in humans were identified 40,41. In a randomized, placebo-controlled study involving ten healthy subjects, Monauni *et al.* 40 assessed the effects of GlcN on insulin secretion and action. An intravenous glucose tolerance test (IVGTT) and a euglycaemic insulin clamp during either a saline infusion or a low (1.6 µmol/min/kg) or high (5.0 µmol/min/kg) GlcN infusion were performed. The authors reported that high-dose GlcN infusion was associated with a modest worsening of glucose tolerance following IVGTT. At GlcN plasma concentrations of approximately 570 and 1150 µmol/L, there was a slight dose-responsive increase in fasting plasma glucose concentration of 0.3 and 0.5 mmol/L, respectively (*p* < 0.05). Plasma glucose concentrations following IVGTT were similarly affected but only at the higher of the two plasma GlcN concentrations. Mechanistically, the authors concluded that the slight vertical upward shift in the plasma glucose concentration curve during high-dose GlcN infusion was likely to be a function of GlcN's well-established capacity to inhibit pancreatic glucokinase 42,43. Further analysis of *beta*-cell function revealed no effect of GlcN on readily reversible, or glucose-stimulated, insulin secretion, suggesting that inhibition of the enzyme was weak at this plasma concentration. At a plasma concentration of 1150 µmol/L, GlcN increased the IVGTT-derived plasma glucose threshold (∼10%; *p* < 0.05), and decreased IVGTT-derived insulin sensitivity and glucose effectiveness (*p* < 0.05). These observations confirm the ability of GlcN, at high concentrations, to inhibit glucokinase activity, which in turn attenuates the glucose-sensing capacity of the *beta* cells and is the cause of the increase in glucose plasma levels under these conditions. No significant changes in glucose tolerance were observed at a lower, though still very high (570 µmol/L), plasma GlcN concentration.

Pouwels *et al.* 41 investigated the effects of intravenous GlcN·SO_4_ on insulin-stimulated forearm skeletal muscle glucose uptake and whole body glucose uptake in 20 healthy normoglycaemic volunteers (10 men and 10 women; mean age 24 ± 4 years; body mass index 22.3 ± 1.9 kg/m^2^). The authors employed a double forearm balance technique (infused arm *versus* control arm), with insulin sensitivity monitored *via* euglycaemic hyperinsulinaemic clamping (insulin 60 mU/m^2^/ min + glucose 20%). During clamping, GlcN was administered to one of the two groups at an infusion rate of 40 µmol/L/min for 150 or 300 min (*n* = 6 per group), and six subjects were infused with saline representing the placebo control. Following GlcN infusion, plasma GlcN concentrations increased to 420 ± 140 µmol/L and to 810 ± 460 µmol/L during the 150- and 300-min infusion periods, respectively. The authors reported that under these conditions, infusion of GlcN into the brachial artery for up to 300 min did not affect total body insulin sensitivity. They concluded that GlcN had no effect on insulin-induced glucose uptake, and that a role for the hexosamine biosynthesis pathway in regulating insulin sensitivity in humans was not supported under their study conditions.

### Oral GlcN supplementation in humans

#### Studies conducted in diabetic or pre-diabetic subjects

In total, six studies were identified from the literature in which GlcN was administered to subjects with confirmed diabetes and or subjects with poor glucose tolerance and apparent insulin resistance 7,15,44–47. Two of these studies were uncontrolled interventions 45,47, and the remaining four studies were controlled interventions consisting of two randomized placebo-controlled investigations 7,10,44,46. These studies involved the administration of GlcN at typical supplemental quantities (1500 mg), for treatment periods of between 1 and 90 days. Three studies conducted glucose tolerance testing and one study measured insulin sensitivity using hyperinsulinaemic–euglycaemic clamping 7,15,45. These studies are described in detail below, and a summary of the fasting glucose concentrations, results of glucose tolerance testing, and clamping analyses are presented in Table 1.

**Table 1:** Summary of glucosamine clinical trials containing diabetes-related endpoints conducted in diabetic or pre-diabetic subjects

					Fasting glucose concentration (mM) Mean (SD)^a^							
								
					Glucosamine		Control					
										
Study	Number of subjects (glucosamine/control)	Dose (mg)	Type of study	Duration (days)	Before	After	Before	After	Net %Δ C-G	Oral glucose tolerance testing	Insulin sensitivity (hyper–insulinaemic–euglycaemic clamping) change	Other diabetes-related endpoints
Biggee *et al.*^7^												
Normoglycaemic	13	1500	Cross-over study	Acute	na	na	na	na	—	No change	—	No effect on 2-h area under the curve insulin profile
Outlier subjects	3				na	na	na	na	—	↑Area under the curve	—	—
Albert *et al.*^44^	12	1500	Randomized placebo-controlled cross-over study	14	9.5 (5.4)	10.3 (4.9)	9.5 (4.2)	10.5 (6.2)	− 2.1%	—	—	—
Diabetes type 1 and 2												
Muniyappa *et al.*^15^												
Lean	20	1500	Randomized placebo-controlled cross-over study -	42	4.5 (0.5)	4.5 (0.2)	4.5 (0.5)	4.6 (0.5)	− 1.6%	—	No change	No effect on quantitative insulin sensitivity check index, fasting insulin, haemoglobin A_1c_ in obese or lean subjects
Obese	20				4.9 (0.5)	4.8 (0.5)	4.9 (0.5)	4.8 (0.5)	0%	—	No change	No effect on endothelial function in obese or lean subjects
Yu *et al.*^45^												No effect on fasting plasma insulin, lipoprotein, or 4-h meal tolerance test plasma insulin area under the curve in obese or lean subjects
Lean	7	1500	Non-controlled intervention	28	4.8 (0.3)	Not clinically significant	—	—	—	No change	No change^b^	
Obese	7				5.4 (0.3)	Not clinically significant	—	—	—	No change	No change^b^	
Scroggie *et al.*^46^	22/12	1500	Randomized placebo controlled	90	na	na	na	na	—	—	—	No significant effect on haemoglobin A_1c_
Tapadinhas *et al.*^47^	1208 diabetes (92)	1500	Non-controlled intervention	50 ± 14	na	na	na	na	—	—	—	Authors reported no effect on tolerability rating among subjects with diabetes

na, not available.

a Studies reporting standard errors were back calculated to obtain standard deviation estimates.

b Conditions of insulin sensitivity testing unclear.

In an uncontrolled study, Yu *et al.* 45 investigated the effects of 4 weeks of oral GlcN·SO_4_ use (1500 mg/day) on insulin sensitivity and glucose response in seven lean and seven obese subjects. Two of the lean and three of the obese subjects displayed impaired glucose tolerance. After 4 weeks of GlcN administration, there were no differences in fasting plasma glucose or insulin levels in either the lean or obese subjects, and the pooled (lean + obese) results from the glucose challenge and insulin sensitivity analyses did not differ between baseline and week 4. Sub-group analyses of the data based on body mass index or glucose tolerance showed no GlcN effect.

The administration of GlcN to diabetic subjects was reported as part of a large multi-centre uncontrolled intervention study carried out by 252 doctors across Portugal 47. In total, 1208 osteoarthritis subjects received GlcN·SO_4_ (1500 mg/day) for 6–8 weeks, and included 92 subjects with reported diabetes, of whom 74 were on hypoglycaemic medication. The authors reported that no variation intolerability was observed among the GlcN users with diabetes, whether they were using hypoglycaemic medication or not.

Four controlled intervention studies investigating the effect of oral GlcN use on diabetes outcomes as primary endpoints were identified 7,15,44,46.

Albert *et al.* 44 investigated the effects of GlcN consumption on a number of diabetes-related endpoints in subjects with type 1 or 2 diabetes. Three females and nine males were enrolled in the study. Two had type 1 diabetes, and ten type 2 diabetes. Subjects were randomized to a double-blind placebo-controlled cross-over study where each subject was administered placebo or GlcN capsules three times daily, resulting in the consumption of 1500 mg of GlcN per day during the treatment interval. The treatment and placebo intervals were 2 weeks in duration separated by a 4-week washout period. Fasting plasma glucose and haemoglobin A_1c_ (HbA_1c_) values were measured serially. There were no statistically significant changes or non-significant trends in any measured parameter following 2 weeks of GlcN treatment relative to baseline or placebo controls. The authors concluded that ‘GlcN at doses commonly consumed does not have significant effects on glycaemic control of diabetic subjects after 2 weeks of supplementation’. Some of the caveats to this study are the small sample size, short duration of treatment, and use of insensitive glucose tolerance and insulin response monitoring.

Evidence of impaired glucose tolerance following GlcN use was suggested by Biggee *et al.* 7 based on observations from three subjects who had outlier results for glucose tolerance tests after GlcN·SO_4_ administration. The authors investigated the effect of GlcN·SO_4_ on glucose tolerance in 16 patients with osteoarthritis who had normal fasting plasma glucose values. The study employed a cross-over study design with treatments allocated in a non-random manner during three visits, 1–2 weeks apart and a final visit 4 months later. For each participant, serum glucose and insulin area under the curve (AUC) values were determined over a 2- and 3-h period, respectively, immediately following the consumption of one of the four treatment regimes: (1) GlcN (1500 mg) only at visit 1; (2) glucose only (75 g) at visit 2; (3) combined GlcN (1500 mg) and glucose (75 g) challenge at visit 3; and (4) a control evaluation in the absence of glucose or GlcN on visit 4. The *post hoc* analysis was based on the results of oral glucose tolerance testing (OGTT) for 3 subjects who had higher serum glucose values with the OGTT compared with the 13 subjects with lower glucose values during the OGTT. One of the three outlier subjects had a normal baseline OGTT but a slightly abnormal value after GlcN consumption. The authors reported that oral GlcN did not affect glucose or insulin AUC values in the 13 normoglycaemic patients; however, in the 3 outlier subjects, the consumption of GlcN (1500 mg) immediately following the oral glucose challenge resulted in a 32% (*p* < 0.05) increase in the glucose AUC values relative to the standard glucose challenge. There was no change in insulin AUC values following a glucose challenge with or without GlcN. It also was noted that subjects were not allocated to treatment groups at random; subjects with poor glucose tolerance tend to worsen over time, and baseline data on fasting glucose values for the three outlier subjects at visit 1 and 4 are not presented, which confounds interpretation of the GlcN effect in these subjects. On the basis of the evidence of worsening glucose tolerance among the outliers, the investigators concluded that oral GlcN may decrease glucose tolerance in subjects with ‘undiagnosed’ diabetes. The limitations of this study are the small numbers of subjects (three in the impaired fasting glucose tolerance group), use of *post hoc* analyses to identify the three outlying ‘responders’, the large variability in glucose tolerance test results, and failure to allocate subjects to the treatments in a randomized fashion.

Muniyappa *et al.* 15 conducted a randomized, double-blind, placebo-controlled cross-over study to assess the effect of 6 weeks of oral GlcN·HCl use (1500 mg/day) on insulin resistance in 20 lean and 20 healthy obese subjects. Insulin resistance was assessed using euglycaemic–hyperinsulinaemic clamp methodology. At baseline, the obese subjects displayed significant insulin resistance (*p* < 0.0001) relative to lean subjects. Therefore, this study allows the effect of GlcN on subjects with and without apparent insulin resistance to be assessed. There were no significant differences between groups for the various diabetes-related analytical endpoints. No within-group differences from baseline were reported. The authors concluded that GlcN neither worsens insulin resistance in lean healthy subjects nor in obese ‘pre-diabetic’ subjects.

Scroggie *et al.* 46 administered GlcN·HCl (1500 mg/day) and chondroitin sulfate (1200 mg/day) to 26 male and female patients with type 2 diabetes for 90 days and 12 others received placebo. Percent HbA_1c_ rose by 0.05% in subjects administered GlcN for 90 days, and was reduced by 0.16% at day 90 in the placebo group. Although the authors reported that the between-group differences were not significant (analysis of variance, *p* = 0.2), it must be noted that the study is underpowered to detect a significant change of the magnitude observed by the authors.

Thus, of the six intervention studies identified, two of which were uncontrolled, deleterious effects of GlcN on glucose metabolism were reported only in one study and only in a *post hoc* analysis involving three ‘outlier’ subjects. The totality of scientific evidence, including a study employing the gold-standard euglycaemic–hyperinsulinaemic clamp methodology, does not indicate any adverse effects of GlcN, at therapeutic doses normally consumed, on glucose metabolism in subjects with poor glucose tolerance or in subjects with type 2 diabetes.

#### Studies conducted in normoglycaemic individuals

The glycaemic response of non-diabetic subjects to GlcN administration has been examined in 13 clinical trials involving 1973 subjects with a duration of exposure from 21 to 1095 days (median, 250 days; Table 2).

**Table 2:** Summary of glucosamine clinical trials containing diabetes-related endpoints conducted in normoglycaemic subjects

					Fasting glucose concentration (mM) Mean (SD)^a^							
								
					Glucosamine		Control					
										
Study	Number of subjects (glucosamine/control)	Dose (mg)	Duration (days)	Type of study	Before	After	Before	After	Net %Δ C-G	Oral glucose tolerance testing	Insulin sensitivity change	Other diabetes-related endpoints
Pham *et al.*^6^	38/0	1500	42	NCI	na	na	na	na	—	—	—	NCS change in haemoglobin A_1c_, or fasting insulin levels homeostasis model assessment of insulin resistance increased at 6 weeks (+20%; *p* = 0.04)
Clegg *et al.*^48^	248/242	1500	168	RPC	na	NCS	na	NCS	—	—	—	Haemoglobin A_1c_ levels monitored in diabetics, no changes noted
Tannis *et al.*^49^	11/8	1500	84	RPC	4.6 (0.6)	4.4 (0.6)	4.2 (0.7)	4.5 (0.8)	− 9.2	No change	—	NCS change in haemoglobin A_1c_, fasting insulin levels, or serum insulin response during oral glucose tolerance test
Hughes and Carr 50	39/39	1500	168	Randomized controlled	na	NCS	na	NCS	—	—	—	—
Pavelka *et al.*^51^	101/101	1500	1095	RPC	na	NCS	na	NCS	—	—	—	Four patients diagnosed with diabetes at end of trial, only one was receiving glucosamine
Reginster *et al.*^52^	106/106	1500	1095	RPC	na	NCS	na	NCS	—	—	—	—
Almada *et al.*^53^	6/9	1500	84	RPC	5.2 (0.5)	5.1 (0.7)	5.2 (0.9)	5.2 (0.7)	− 1.9	—	—	NCS change in fasting insulin relative to baseline. Δ in fasting insulin significantly different between groups (P: 1.1 *versus* GS: 4.9 ± 4.3; *p* = 0.01). Δ in fasting insulin resistance index significantly different between groups (*p*: − 0.34 ± 0.9 *versus* 1.0 ± 1.0; *p* = 0.01).
Giordano *et al.*^54^	20/0	1500	365	NCI	na	NCS	—	NCS	—	—	—	—
Noack *et al.*^55^	126/126	1500	28	RPC	na	NCS	na	NCS	—	—	—	—
Tapadinhas *et al.*^47^	1208 Diabetes (92)	1500	50 ± 14	NCI	na	na	na	na	—	—	—	—
D'Ambrosio *et al.*^56^	15/15	1500	21	Randomized controlled	6.1 (1.2)	5.4 (0.1)	5.8 (0.1)	5.3 (0.1)	− 3.4	—	—	—
Drovanti *et al.*^57^	40/40	1500	30	RPC	4.6 (0.06)	4.6 (0.06)	4.4 (0.13)	4.4 (0.13)	0.0	—	—	—
Crolle and D'este 58	15/15	1500	21	Randomized controlled	5.3 (0.16)	5.7 (0.13)	4.7 (0.07)	5.4 (0.13)	− 7.4	—	—	—
Laferrère *et al.*^59^	6/9	3000	acute	NCI	—	NCS	—	NCS	—	—	—	NCS change in fasting insulin
	5/9	6000		NCI	—	NCS	—	NCS	—	—	—	—
All net change										− 3.7		

na, not available; NCI, non-controlled intervention study; NCS, not clinically or statistically significant; RPC, randomized placebo controlled.

a Studies reporting standard errors were back calculated to obtain standard deviation estimates.

In an uncontrolled intervention study, Pham *et al.* 6 reported that ‘oral GlcN in doses used to treat osteoarthritis worsens insulin resistance’. This study was conducted in 38 male and female subjects in the age group of 23–67 years with an average age of 43.9 years. The treatment consisted of GlcN administration (1500 mg; type not stated) once daily for a period of 42 days. On days 0 and 43, subjects had blood sampled to determine fasting blood glucose, insulin, and serum lipid levels. Fasting glucose and insulin levels were used to calculate a homeostasis model assessment of insulin resistance and quantitative insulin sensitivity check index quantitative insulin sensitivity check index. Unfortunately, data on fasting plasma glucose values (mean and range) at baseline and final visits are not provided. After consuming GlcN for 42 days, the mean post-trial body mass index, fasting insulin, and HbA_1c_ level were not significantly different from day 0. Thirty-eight subjects were enrolled in the study but the number of subjects included at each endpoint analysis varied substantially and no details of the number of subjects completing the study, and subjects unavailable for specific endpoint analyses were provided. The authors reported that evidence of insulin resistance was observed in the group post-treatment as the homeostasis model assessment of insulin resistance values were increased by 20% from baseline (*n* = 35; *p* = 0.04). HbA_1c_ values decreased, but non-significantly, during the study from 5.54 ± 0.74 at baseline to 5.45 ± 0.51 at day 43, an observation that is inconsistent with the authors' conclusion that glucose tolerance worsened in subjects with poorer insulin sensitivity. Several additional *post hoc* correlations between baseline measurements of homeostasis model assessment relative to changes in quantitative insulin sensitivity check index, low-density lipoprotein, and small artery elasticity are presented as worsening over time in subjects with higher baseline homeostasis model assessment values, an effect the authors attribute to GlcN. Because of the unavailability of vital fasting plasma glucose data, failure to report data on intent-to-treat basis, use of *post hoc* comparisons, and uncontrolled study design the authors' conclusion are not supported.

The recent GlcN/Chondroitin Arthritis Intervention Trial commissioned by the National Institutes of Health (United States) investigated the safety of GlcN·HCl use in a large number of osteoarthritic subjects (average age 59 years). The study utilized a randomized double-blind placebo-controlled design and evaluated the exposure to GlcN supplementation over a 6-month period 48. A total of 242 subjects were randomized to receive GlcN treatment (1500 mg/day) and 313 to the placebo group. The study included diabetic subjects (number not reported), who had their fasting plasma glucose or HbA_1c_ monitored during the study. There were no significant GlcN-induced changes in these parameters on conclusion of the study nor was there an increase in cardiovascular disease risk factors in the diabetic patients who received GlcN.

In a double-blind placebo-controlled study by Tannis *et al.* 49 conducted in 19 healthy male and female subjects, daily GlcN·SO_4_ (1500 mg) for 12 weeks had no effect on fasting glucose or insulin levels and no change in glucose tolerance following glucose challenge. Changes in fasting plasma glucose levels in the placebo- (P) and GlcN-treated (GlcN) groups after 6 weeks were + 6.6% (P) and − 15.8% (GlcN), respectively, with net difference, − 22.4 favouring GlcN. After 12 weeks, the corresponding figures were + 5.4% (P) and − 3.7% (GlcN) with net difference, − 9.2% favouring GlcN.

The effects of acute high-dose oral GlcN administration were investigated by Laferrère *et al.* 59 in 20 healthy, non-obese subjects with normal glucose tolerance. Six subjects received 3000 mg of GlcN·SO_4_, five received 6000 mg of GlcN and nine received an inert placebo in the morning following an overnight fast. Neither of the high doses of oral GlcN affected plasma glucose or insulin levels.

A preliminary report—published only as an abstract—was identified in the literature in which oral GlcN·SO_4_ was reported to adversely affect glucose metabolism 53. This was a secondary analysis of a study investigating the effect of GlcN on back pain in which the effects of GlcN on glucose metabolism was evaluated in 15 subjects (6 GlcN, 9 placebo) consuming GlcN (1500 mg) or placebo treatments for a period of 12 weeks. There were no differences between groups in fasting plasma glucose or insulin levels at week 12 but there was a significant (*p* < 0.01) increase in fasting plasma insulin level relative to baseline in those receiving GlcN.

Finally, a number of additional studies (Table 2) were identified during the literature search in which glucose measurements were obtained and/or clinical chemistry monitoring was conducted 50,54–58,60–62. No evidence of GlcN-related effects on fasting glucose concentrations were reported by the authors.

#### Long-term GlcN use

The long-term safety of GlcN has been evaluated in two studies for which diabetes-related endpoints were monitored as secondary endpoints 51,52. Pavelká *et al.* 51 conducted a double-blind placebo-controlled study that randomized 202 subjects with osteoarthritis of the knee to receive either daily placebo or GlcN·SO_4_ treatment (1500 mg) for a period of 3 years. The mean age of subjects randomized to the placebo and control groups was 64 and 61 years, respectively. Standard secondary safety endpoints involved adverse event reporting and routine laboratory testing was performed on a yearly basis. During the study, four patients developed diabetes; three of them were from the placebo group and one from the GlcN group. No GlcN-related effects were reported for the results of the yearly laboratory monitoring, which presumably included fasting plasma glucose levels.

Reginster *et al.* 52 evaluated the effect of GlcN·SO_4_ supplementation in 212 male and female subjects with osteoarthritis of the knee. Participants (mean age of 66 years) were randomized to receive either placebo or GlcN·SO_4_ (1500 mg/day) for a period of 3 years. Diabetes-relevant parameters were monitored as secondary endpoints and, in addition to standard adverse event and laboratory monitoring, each subject had fasting glucose concentrations measured yearly. Drop-out rates were equal in both groups throughout the trial, and reasons for dropping out did not differ. Routine laboratory testing did not show any significant changes in glycaemic homeostasis although fasting plasma glucose levels tended to decrease from baseline in the GlcN-treated group. In a personal communication, the lead author explained that only subjects with diagnosed type 2 diabetes were excluded from the study and that sub-group analysis of the subjects who were randomized to the GlcN group and had above ‘normal’ fasting glucose concentrations at the beginning of the trial displayed trends (non-significant) towards lower plasma glucose concentration throughout the 3 years of daily GlcN use (Reginster, personal correspondence, 2009).

As the average age of the subjects in these two studies was above 60 years, a significant proportion of the patients who participated in these studies might have been expected to have some degree of glucose intolerance, the prevalence of which has been estimated by some to be as high as 52% for Europeans 60–79 years of age 63. On the basis of data collected from the International Diabetes Federation 64 and European census data 65, it has been estimated that 17% of people recruited between the ages of 60 and 79 from the Belgian population 52 and 23% of those from the Czech populations 51 would have evidence of impaired glucose tolerance, and might therefore be considered potentially pre-diabetic. Although some of these potentially ‘pre-diabetic’ subjects might have been excluded from each of the two long-term clinical trials (e.g. obese subjects) it is not unreasonable to suppose, given its high prevalence, that a significant number of individuals with impaired glucose tolerance would have been included in these studies of long-term GlcN administration.

Although, to date, no study has specifically addressed the long-term safety of GlcN in diabetic or pre-diabetic subjects, the lack of diabetes-related adverse effects, or increases in the incidence of diabetes among 207 elderly subjects receiving daily GlcN supplementation for a total period of 1242 patient years of follow-up suggest that GlcN does not constitute a specific risk for diabetes.

Overall, data obtained from 12 clinical trials, including 765 non-diabetic subjects receiving standard clinical doses of GlcN (1500 mg) for periods of up to 3 years have not suggested adverse effects on glycaemia or the progression/development of diabetes in older volunteer subjects (Table 2). Even when infused at high doses of up to 269 mg/kg body weight, resulting in plasma levels of 187-fold above those expected under standard oral dosing, no effects on insulin resistance are observed.

## Discussion

**Figure 1:**
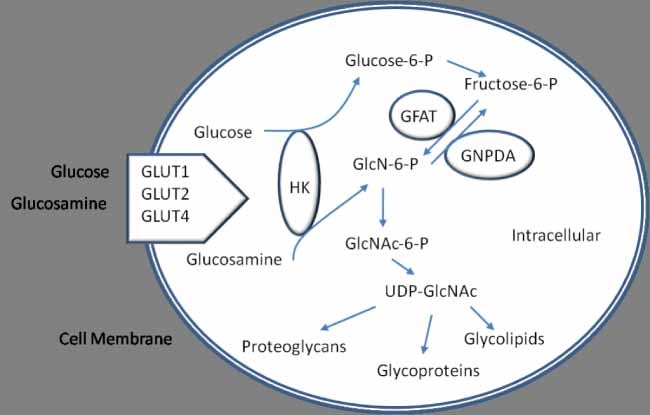
Glucosamine and the hexosamine biosynthesis pathway. Glucose transporters are indicated by arrow and major enzymes are included in ellipses. GLUT1, GLUT2, GLUT4, glucose transporters; GlcN-6-P, glucosamine-6-phosphate; Glc-NAc-6-P, *N*-acetyl-glucosamine-6-phosphate; UDPGalNAc, uridine diphosphate-*N*-acetyl-galactosamine; UDP-GlcNAc, UDP-*N*-acetyl-glucosamine; HK, hexokinase; GFAT, glucosamine: fructose-6-phosphate aminotransferase; and GNPDA, glucosamine-6-phosphate deaminase

GlcN is a widely used dietary supplement that is described as efficacious and safe for many individuals with osteoarthritis, especially of the knees 66. Concerns that GlcN consumption may worsen glucose tolerance and induce insulin resistance were not based on clinical observations, but on *in vitro* studies by Marshall *et al.* showing that exogenous GlcN could increase the activity of the hexosamine biosynthesis pathway, a metabolic process that is believed to function as a nutrient sensor modulating insulin sensitivity and glucose uptake in peripheral tissues 4 (Figure 1). The end product of this pathway is UDP-*N*-acetylGlcN, a substrate for *O*-GlcNAc transferase, which mediates the addition of β-*N*-acetylGlcN to the hydroxyl groups of serine and/or threonine residues on a wide variety of proteins. This post-translational modification regulates a wide range of biological processes, including signal transduction/metabolic proteins that modulate glucose metabolism and insulin sensitivity 5. As reviewed by Copeland *et al.* 5, there are strong associations between elevated GlcN acylation of proteins with glucose toxicity and impaired insulin signalling; excessive flux of sugars through the hexosamine signalling pathway has therefore been implicated as a causative factor in the development of type 2 diabetes.

There is a large body of evidence from *in vitro* studies using human- and rodent-derived cells that high concentrations of GlcN in the incubation media impairs insulin-mediated glucose uptake. This has led mistakenly to GlcN being described as a diabetogenic agent. Interference with glucose metabolism occurs only at concentrations comparable with those of glucose, i.e. within the 2–50 mmol/L range, concentrations that are several hundred- to a thousand-fold greater than plasma concentrations that occur during oral supplement use. At these concentrations (<10 µmol/L), GlcN neither augments the hexosamine biosynthesis pathway nor reduces insulin-mediated glucose uptake 67,68. Additionally, *in vitro* observations in human adipocytes have shown that in the presence of normal glucose concentrations, the uptake of GlcN is almost completely inhibited 67.

Animals-administered high intravenous concentrations of GlcN have shown that rodents are especially sensitive to its diabetogenic effects. During euglycaemic–hyperinsulinaemic clamping, the infusion of GlcN in rodents producing plasma GlcN concentrations of between 800 and 1200 µmol/L results in glucose intolerance and insulin insensitivity 32,37. In contrast, the consumption of GlcN at doses many times greater than are used clinically was not associated with any adverse effects on glucose metabolism. For example, Echard *et al.* 69 reported that in strains of rodents that are highly sensitive to sugar-induced insulin resistance, the consumption of GlcN in large amounts (9% in the diet; ∼4.5 g/kg body weight) for 9 months had no effect on fasting glucose concentrations or glucose tolerance. Similar observations have been made in other species (dog and rabbit) in which GlcN was administered orally, at doses greatly exceeding those used clinically 70,71. In none of these studies were sensitive insulin response monitoring methods employed or plasma GlcN levels measured; nevertheless, the absence of a diabetogenic effect in animal feeding studies is consistent with its low bioavailability and its lack of biological effect on glucose metabolism.

In clinical trials investigating the effect of GlcN on osteoarthritis, GlcN has been administered to many patients with type 2 diabetes or impaired glucose tolerance, without specifying the numbers. In long-term randomized placebo-controlled studies, daily GlcN use was not associated with increased incidences of type 2 diabetes, and a non-significant fall in fasting blood glucose values over 3-year periods of daily GlcN use was reported in one study, an effect that could be explicable by the concept of regression to the mean as it was observed only in those with the highest blood glucose values at the start 51,52. Overall, the data from randomized placebo-controlled osteoarthritis trials have not shown any adverse effects on fasting blood glucose levels, glucose metabolism, or insulin sensitivity from oral GlcN, at any dose level.

Six studies that investigated diabetes-related outcomes as primary endpoints were identified, and included participants with type 2 diabetes or obese subjects with apparent insulin resistance 7,15,44–46. In most short-term studies, there were no significant changes in fasting plasma glucose or insulin concentrations or in HbA_1c_ after consumption of GlcN in usual doses. Nor was there any change in insulin sensitivity determined by the use of a hyperglycaemic clamp. One short-term clinical study 7, which concluded that oral GlcN use in ‘pre-diabetic’ subjects may adversely affect insulin sensitivity and glucose tolerance, is flawed by its small sample sizes (*n* = 3), the use of *post hoc* analysis, failure to allocate subjects to treatment groups in a randomized fashion, and the inherent limitations of uncontrolled intervention studies.

The only diabetogenic effect attributed to GlcN in humans was in response to high-dose GlcN infusion, which resulted in a slight elevation in plasma glucose concentrations following an IVGTT 40. However, this observation was not due to the development of insulin resistance, and was consistent with GlcN's well-established inhibitory activity towards glucokinase, an effect that in humans would only occur when plasma levels of GlcN approach normal glucose concentrations. This concentration (5 mM) is roughly 500- to 1000-fold above plasma levels that are reasonably expected following supplemental GlcN use.

In conclusion, the available evidence implicating GlcN as a diabetogenic agent are limited to rodent infusion studies and *in vitro* observations, experimental models that were determined not to be relevant to humans. A comprehensive and critical review of the clinical literature indicated that the consumption of GlcN at usual doses was well tolerated by normal, diabetic, and ‘pre-diabetic’ subjects. Thus, based on the overall weight of scientific evidence, there currently appears to be no reason to restrict the use of oral GlcN for individuals at risk for diabetes, or those with type 1 or 2 diabetes, or normoglycaemics with respect to any adverse effects on sugar metabolism.

## Conflict of interest

This review was supported in part by Cargill Incorporated, Eddyville, IA, a manufacturer of GlcN. R. Simon is an employee of Cantox Health Sciences International, and financial support from Cargill for consulting services has been obtained. Drs V. Marks and J.W. Anderson are consultants to Cargill, Inc. Dr A.R. Leeds has declared no conflicts of interest.
